# The effect of comprehensive intervention on family support and the mediating effect between intervention and changes in children’s dietary and physical activity behaviors

**DOI:** 10.1371/journal.pone.0339009

**Published:** 2026-01-22

**Authors:** Peifen Duan, Chen Li, Songyang Yu, Jianhui Yuan, Xiangxian Feng

**Affiliations:** 1 School of Public Health, Changzhi Medical College, Changzhi, China; 2 Shanxi Higher Education Institutions of Science and Technology Innovation Plan Platform, Laboratory of Environmental Factors and Population Health, Changzhi, China; 3 Key Laboratory of Environmental Pathogenic Mechanisms and Prevention of Chronic Diseases, Changzhi Medical College, Changzhi, China; 4 School of Nursing, Changzhi Medical College, Changzhi, China; University of Maribor, SLOVENIA

## Abstract

**Objective:**

Childhood overweight and obesity have become one of the major global public health problems. Family support plays an important role in the development of children’s healthy behaviors. This study aimed to assess the effect of a comprehensive intervention for childhood obesity on family support and to examine the mediating effect of family support between the intervention and changes in children’s dietary and physical activity behaviors.

**Methods:**

A cluster-randomized controlled trial was conducted from 2018 to 2019, involving 396 students from eight elementary schools in Changzhi City, China. Data on children’s dietary habits, physical activity, and family support were collected via structured questionnaires. Generalized estimating equation models were used to assess the effects of the intervention on family support and its mediating effects were examined.

**Results:**

After one academic year, the intervention group demonstrated a higher level of family support compared to the control group (OR = 2.539, 95% CI: 2.163–5.402), with increases observed from fathers (OR = 1.910, 95% CI: 1.620–4.087), mothers (OR = 3.624, 95% CI: 1.826–4.092), and grandmothers (OR = 1.289, 95% CI: 1.009–1.648). Mediation analysis indicated that changes in family support partially mediated the association between the intervention and improvements in children’s dietary habits and screen time.

**Conclusions:**

The comprehensive intervention effectively enhanced family support, and changes in family support served as a mediator for the improvements in children’s dietary behaviors. Integrating family based strategies into the comprehensive childhood obesity prevention programs may further enhance intervention efficacy.

## Introduction

The rapid socioeconomic development and shifts in dietary patterns have contributed to increasingly imbalanced nutrition and physical inactivity among children, exacerbating the global epidemic of childhood overweight and obesity [1–3].

It is estimated that approximately 435 million children and adolescents aged 5–19 years are affected by overweight or obesity globally. If current trends continue, the prevalence is projected to reach 40% by 2035, affecting approximately 770 million young people [4]. Childhood overweight and obesity have become an important public health problem, especially in developing countries [5]. According to a national report, the prevalence rates of overweight and obesity among children and adolescents aged 6–17 years in China reached 11.1% and 7.9%, respectively [6].

Childhood overweight and obesity are associated with a range of serious health consequences, including psychological sequelae such as diminished self-esteem and anxiety, as well as potential impairments in social competence and interpersonal functioning [7–10]. Childhood overweight and obesity have been linked to a range of determinants including genetic predisposition, dietary pattern shifts, physical inactivity, prolonged screen time, and insufficient sleep [11–13]. As the primary socialization environment, the family plays a pivotal role in shaping children’s perceptions of lifestyle and health behaviors. A supportive family environment not only promotes the development of healthy habits but also enhances overall physical and psychological well-being [14–16]. Several studies have demonstrated that family support and education significantly improve children’s academic performance, lifestyle choices, and physical health [17–19]. However, there is a scarcity of research on the role of family support in interventions targeting children’s diet and exercise behaviors, and the results are inconsistent [20–23]. Furthermore, different family members may play distinct roles in shaping children’s dietary and physical activity behaviors.

This study therefore aimed to investigate the effect of a comprehensive intervention on family support and to elucidate its mediating role in improving children’s diet and physical activity, while also delineating the specific contributions of different family members.

## Materials and methods

### Study design and participants

**Fig 1:**
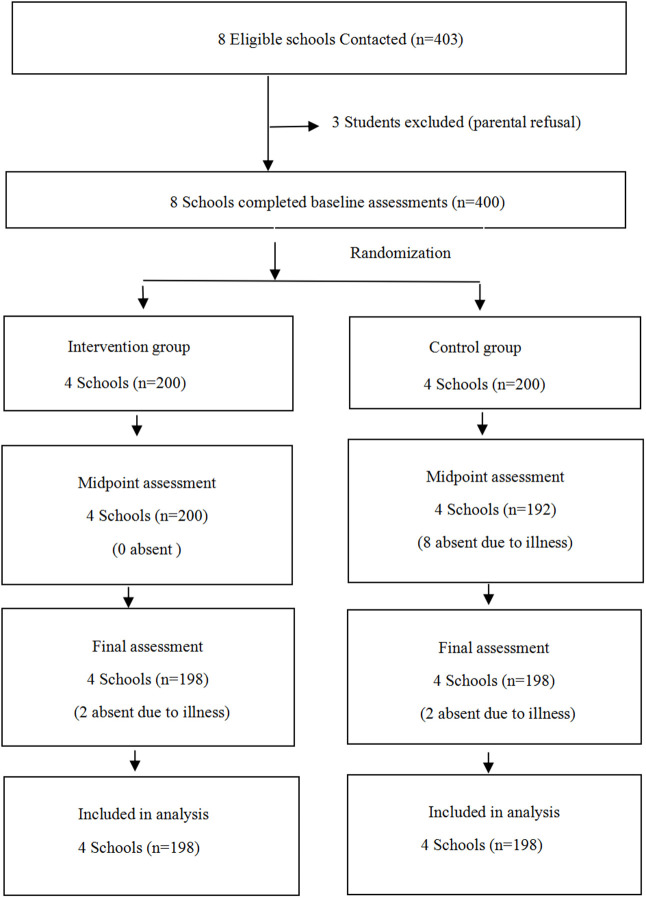
Participant enrollment and allocation flowchart.

We conducted a school-based cluster randomized controlled trial in Changzhi, Shanxi Province of China. Using a random cluster sampling method, we selected eight primary schools and randomly chose one fourth-grade class from each school as study participants (recruitment period: May 15 to June 15, 2018). Following baseline surveys, we distributed the schools equally between intervention or control arms (four per condition). Randomization was generated using a centralized computer, and an independent statistician maintained the concealed assignment sequences.The comprehensive intervention was conducted over one academic year, from September 1, 2018, to June 30, 2019. (see S1 File.) The study population comprised 396 students (198 per group) who completed both the baseline and follow-up assessments after one academic year of intervention (Fig 1). The study protocol was approved by the Biomedical Ethics Committee of Peking University (Approval No: IRB00001052–18021). Written informed consent was obtained from all participating children and their parents/legal guardians before data collection. This trial was registered at the Chinese Clinical Trial Registry (registration number: NCT03665857)

### Description of intervention

Based on the socio-ecological model [24], this study implemented a one academic year intervention targeting children’s diet and physical activity in the intervention group. The intervention consisted of five components, three of which were aimed at children and mainly included (1) Health education activities: The health education activities were conducted by trained teachers. The core messages included not overeating, not drinking sugar-sweetened beverages, eating less energy-dense foods, reducing sedentary time, and increasing physical activity. (2) Physical activity: Daily 60-minute sessions of moderate-to-vigorous physical activity (MVPA) were conducted under the supervision of certified physical education teachers. (3) Monitoring and feedback of height and weight: Height and weight were assessed monthly to calculate Body Mass Index (BMI). Following each assessment, individualized feedback on measurement trends was provided to families through a mobile application. The other two environmental components targeted both school and home settings. School-level interventions included working with principals to develop health-promoting policies, such as banning the sale of unhealthy snacks and sugary drinks. At the same time, parents were given health education and instructed to monitor their children’s dietary behavior. Schools assigned to the control group maintained their usual health and physical education programs. A comprehensive summary of the intervention components is presented in S1 Table.

### Data collection

Before and after the intervention, physical examinations and questionnaire surveys were conducted by uniformly trained investigators. The staff who implemented the interventions did not participate in the follow-up assessments. Key measurements included anthropometric measurements (height and weight), which were used to calculate BMI. The questionnaire covered: demographic characteristics (gender, age); knowledge related to diet and physical activity; dietary and physical activity behaviors and attitudes, and family members’ supportive attitudes toward the intervention.

### Definition of relevant obesity measures

#### Weight status.

Weight status was classified according to China’s WS/T 586–2018 “screening for overweight and obesity in school-age children and adolescents” standard [25]. Participants were categorized into two groups: a non-overweight/obesity group (including underweight and normal weight) and an overweight/obesity group (including overweight and obesity).

#### Family support.

Primary caregivers, including parents and grandparents, were surveyed via questionnaire to assess their supportive attitudes toward the intervention. (1) Family support attitude: Participants who selected “support” were classified as supportive; all other responses (objection, unclear, and indifferent) were dichotomized as non-supportive. (2) Family support assessment: Family support was defined as endorsement from at least one family member. Conversely, its absence was recorded when no family member expressed support.

#### Diet and exercise related cognition and behaviors.

The cognition and behavior related to diet and exercise were evaluated through four dimensions: (1) Diet and exercise knowledge, calculated as the rate of correct responses; (2) Dietary habits, evaluated by the avoidance of snacks, fried foods, and Western fast food; (3) Physical activity, measured as days per week achieving ≥60 minutes of MVPA; (4) Screen time, defined as an average of less than 2 hours per day. According to the median of baseline levels, children’s diet and exercise knowledge, dietary habits, and physical activity were classified as high or low, where higher scores indicated better knowledge, healthier habits, and more frequent exercise. Screen time was classified as high or low according to whether the average screen time was ≤ 2 hours, with high representing shorter screen time.

### Sample size

The sample size was determined a priori to be 400 participants, based on a two-sided alpha of 0.05, 88% power, an effect size of 0.50 BMI units (standard deviation (SD)=1.40), and inclusion of a 10% attrition rate. This was achieved by recruiting an average of 50 students per school cluster.

### Statistical analysis

**Fig 2:**
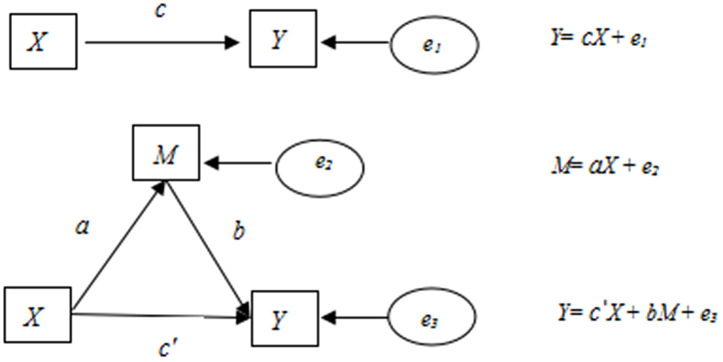
Model of the mediating effect pathway.

All statistical analyses were performed using SPSS 26.0 and R 4.4.0 software. Categorical variables were summarized as frequencies and percentages, with group comparisons conducted using Chi-square tests. Continuous variables with normal distribution were presented as mean ± standard deviation, and intergroup differences were assessed via independent samples t-tests. We employed a generalized estimating equation (GEE) model to evaluate the effect of dietary and physical activity intervention on family support (overall family support and specific family member support) and the mediating role of family support between the intervention and the changes in child outcomes (dietary and exercise knowledge, dietary habits, physical activity, and screen time). All models were adjusted for sex and baseline age and accounted for within-school clustering. As illustrated in Fig 2, path c represents the total effect of the independent variable (X) on the dependent variable (Y). After adjusting for the mediator (M), path c’ represents the direct effect of X on Y. The indirect effect of X on Y through M was quantified by the product of the coefficients a and b (a × b) [26]. In this study, the intervention was used as the independent variable (X), the changes in child outcomes were used as the dependent variable (Y), and the changes in family support were used as the mediating variable (M) to conduct the mediating effect analysis. Statistical significance was set at a two-sided P < 0.05. P-values for the exploratory analyses of specific family members were reported unadjusted for multiple comparisons.

## Results

### Baseline characteristics of the study subjects

A total of 400 students from eight schools were initially enrolled and completed the baseline assessment. However, four students (two per group) were lost to follow-up during the study due to illness. Ultimately, the trial included 396 participants, with 198 children in the intervention group and 198 in the control group. The intervention group comprised 89 boys (44.95%) and 109 girls (55.05%), while the control group had 109 boys (55.05%) and 89 girls (44.95%). Baseline comparisons showed no significant differences between groups in family support, weight status, or screen time (all P > 0.05), but significant differences in gender and age (both P < 0.05) (Table 1).

**Table 1:** Baseline characteristics of study subjects.

Variable	Intervention group (n = 198)	Control group (n = 198)	χ^2^/t value	P value
Age (mean ± SD, years)	9.18 ± 0.42	9.10 ± 0.33	2.253	0.025
Gender, n(%)				
Male	89(44.95)	109(55.05)	4.040	0.044
Female	109(55.05)	89(44.95)		
Family support, n(%)				
Overall family support	153(77.27)	162(81.82)	1.257	0.262
Maternal support	114(57.58)	157(79.29)	3.262	0.071
Paternal support	118(59.60)	136(68.69)	2.307	0.129
Grandfather support	57(28.79)	63(31.82)	1.502	0.22
Maternal grandfather support	36(18.18)	27(16.64)	0.194	0.66
Grandmother support	54(27.27)	68(34.34)	1.119	0.29
Maternal grandmother support	53(26.77)	38(19.19)	1.946	0.163
Weight status, n(%)				
Non-overweight/obese	122(61.62)	111(56.06)	1.841	0.606
Overweight/obese	76(38.38)	87(43.94)		
Screen time, n(%)				
High(≤2h/day)	119(60.10)	126(63.64)	0.525	0.469
Low(>2h/day)	79(39.90)	72(36.36)		

### Impact of the diet-physical activity intervention on family support

After one academic year of intervention, the overall family support rate in the intervention group increased by 6.06% (from 77.27% to 83.33%), while the control group showed an 18.19% decrease (from 81.82% to 63.63%), with the intervention group demonstrating significantly greater improvement compared to controls (OR = 2.539, 95% CI: 2.163–5.402, P < 0.001). Specifically, the proportion of support increased significantly among fathers (OR = 1.910, 95% CI: 1.620–4.087, P < 0.001), mothers (OR = 3.624, 95% CI: 1.826–4.092, P < 0.001), and grandmothers (OR = 1.289, 95% CI: 1.009–1.648, P = 0.047), whereas there were no significant differences in support from grandfathers, maternal grandfathers and maternal grandmothers (all P > 0.05) (Table 2).

**Table 2:** Effects of the comprehensive intervention on family support.

Support category	Intervention group (n = 198)		Control group (n = 198)		OR (95%CI)	P value
	Baseline	End			Baseline	End
Overall family support (n,%)	153 (77.27)	165 (83.33)	162 (81.82)	126 (63.63)	2.539 (2.163–5.402)	<0.001
Specific Family Member Support (n,%)						
Maternal	114 (57.58)	155 (78.28)	157 (79.29)	108 (54.55)	3.624 (1.826–4.092)	<0.001
Paternal	118 (59.60)	132 (66.67)	136 (68.69)	87 (43.94)	1.910 (1.620–4.087)	<0.001
Grandfather	57 (28.79)	31 (15.66)	63 (31.82)	32 (16.16)	1.431 (0.672–3.045)	0.647
Maternal grandfather	36 (18.18)	20 (10.10)	27 (16.64)	29 (14.65)	1.042 (0.831–1.115)	0.247
Grandmother	54 (27.27)	61 (30.81)	68 (34.34)	40 (20.20)	1.289 (1.009–1.648)	0.047
Maternal grandmother	53 (26.77)	45 (22.73)	38 (19.19)	34 (17.17)	1.276 (0.959–3.698)	0.092

### Mediating role of family support in the intervention’s impact on children’s cognition and behavior

**Fig 3:**
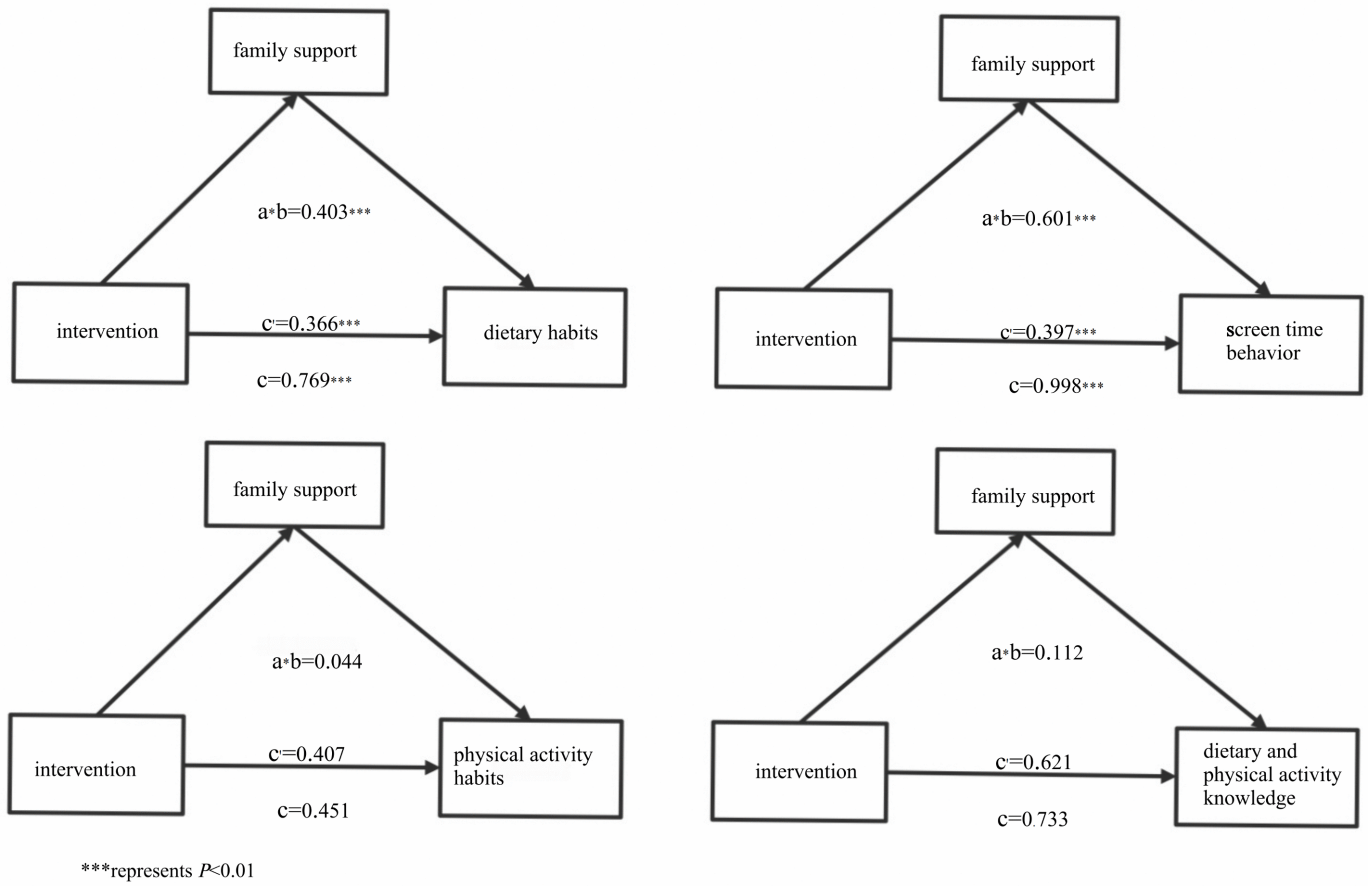
Analysis of the mediating effect of family support.

The mediation analysis revealed that family support mediated the intervention’s effects on children’s dietary habits and screen time (all P < 0.01). Support from fathers, mothers, and maternal grandmothers demonstrated significant mediating roles in these behavioral changes (all P < 0.01), while other family members showed no significant mediation effects. However, family support did not mediate the intervention’s impact on physical activity behaviors or dietary and physical activity knowledge (both P > 0.05) (Tables 3– and Fig 3).

**Table 3:** The mediating role of family support in the association between intervention and children’s dietary habits.

Support type	Total effect	Direct effect	Indirect effect	P value	Effect ratio (%)
Overall family support	0.769	0.366	0.403	<0.010	52.41
Paternal support	0.501	0.286	0.215	<0.010	42.91
Maternal support	0.038	0.017	0.021	<0.010	55.26
Grandfather support	0.25	0.16	0.09	0.132	36.00
Grandmother support	0.091	0.061	0.03	0.527	32.97
Maternal grandfather support	0.245	0.19	0.055	0.417	22.45
Maternal grandmother support	0.433	0.26	0.173	<0.010	39.95

**Table 4:** The mediating role of family support in the association between intervention and screen time.

Support type	Total effect	Direct effect	Indirect effect	P value	Effect ratio (%)
Overall family support	0.998	0.397	0.601	<0.010	60.22
Paternal support	0.525	0.332	0.193	<0.010	36.76
Maternal support	0.645	0.277	0.368	<0.010	56.59
Grandfather support	0.380	0.339	0.041	0.232	10.49
Grandmother support	0.424	0.359	0.065	0.315	15.33
Maternal grandfather support	0.245	0.190	0.055	0.542	22.45
Maternal grandmother support	0.664	0.514	0.150	<0.010	22.59

**Table 5:** The mediating role of family support in the association between intervention and children’s dietary knowledge/physical activity behaviors.

Variables	Total effect	Direct effect	Indirect effect	P value	Effect ratio (%)
Dietary and physical activity knowledge	0.733	0.621	0.112	0.204	15.27
Physical activity habits	0.451	0.407	0.044	0.313	9.75

## Discussion

This randomized controlled trial demonstrated that following one academic year of comprehensive intervention, children in the intervention group showed improved family support compared with the control group. Mediation analyses further indicated that improvements in family support may mediate the intervention’s effects on dietary habits and screen time, but not on physical activity or knowledge related to diet and exercise.

Our comprehensive intervention resulted in a significant improvement in family support. Participants in the intervention group showed greater improvement compared with the control group (OR = 2.539, 95% CI: 2.163–5.402, P < 0.001), consistent with the findings of Haines et al [27], suggesting that the comprehensive intervention can effectively improve the level of family support. Further analysis regarding support from specific family members showed increased supportive behaviors from fathers (OR = 1.910, 95% CI: 1.620–4.087, P < 0.001), mothers (OR = 3.624, 95% CI: 1.826–4.092, P < 0.001), and grandmothers (OR = 1.289, 95% CI: 1.009–1.648, P = 0.047). This pattern of disparity reflects the sociocultural construction of health responsibility allocation within families. The strongest intervention effect observed in mothers aligns with their traditional role as primary caregivers in child health management [28]. Within cultural contexts where multi-generational co-residence remains common, grandmothers maintain substantial involvement in child care and represent a potentially significant influence on health behaviors. Meanwhile, the notable intervention effect observed in fathers coincides with broader socioeconomic trends including the prevalence of dual-income households and evolving societal expectations of fatherhood. Our intervention likely facilitated paternal engagement by providing structured, concrete pathways for participation in child health management.

Families, as the primary environment for child development, play a crucial role in shaping their behavioral habits [29,30]. Unhealthy childhood eating patterns are frequently linked to family meal practices, such as watching television during meals or using food as a reward. Conversely, active parental engagement in children’s nutritional choices and physical activity has been shown to effectively promote healthier eating habits and increase exercise frequency. All this evidence indicated that family support significantly influences children’s dietary and physical activity behaviors [31–35]. However, some studies believed that there was insufficient evidence for the effect of family support on children’s diet and exercise behavior [36,37].

The study analyzed the mediating role of family support between the intervention and children’s cognitive behavioral outcomes. The results suggested that family support mediated the improvement of both dietary habits and screen time, indicating that adequate family support may facilitate the development of healthy behaviors in children, which was consistent with existing evidence [38,39]. The study further revealed that support from fathers, mothers, and maternal grandmothers may mediate the intervention’s effects on children’s dietary habits and screen time. The reason may be related to the child-rearing patterns for school-age children in the studied population. As children’s dietary intake and screen use predominantly occur within the household, these behaviors are strongly influenced by primary caregivers (such as mothers and grandmothers) who oversee daily meal preparation and family routines [40]. Additionally, children’s media habits often model those of their primary caregivers [41,42]. It is suggested that parents should be incorporated into children’s diet and physical activity intervention programs within specific sociocultural contexts. Health education content should provide supportive materials tailored to the distinct perspectives of different family members, which will facilitate the improvement of children’s related cognition and behaviors. However, no mediation effects were found for physical activity or knowledge related to diet and exercise. This may be attributed to the predominant acquisition of cultural knowledge and engagement in physical activities within school settings for school-age children, with relatively little family influence in these aspects.

### Strengths and limitations

Our study has several strengths. First, a multi-component intervention that included health education, structured physical activity, BMI monitoring with feedback, and family engagement was implemented to improve its effectiveness. Second, the study design combined randomization after baseline assessment with blinding of outcome assessors to reduce the potential for selection and measurement bias. Third, the low attrition rate during the study minimized attrition bias. Fourth, the intervention was based on a well-established theoretical framework, which contributed to its structured design and potential efficacy. However, several limitations must be acknowledged. First, while the cluster-randomized design enhances causal inference, all data were self-reported and thus subject to recall and social desirability biases. Second, this study focused exclusively on family support and did not account for other pertinent environmental factors, such as school climate or peer influence. This narrow scope limits the comprehensiveness of the analysis regarding behavioral determinants and may result in an overestimation of the role of family support. Our definition of family support was based on attitudinal measures rather than theoretical classification systems [43], which may not fully capture the spectrum of actual supportive behaviors enacted within the family environment. Third, although the one academic year follow-up period is sufficient for assessing the immediate outcomes of the intervention, it falls short in evaluating the long-term sustainability of behavioral changes and the persistent influence of family support mechanisms. Fourth, the mediation analyses relied on significance testing of the coefficient product, which may limit statistical power and precision. Finally, conducting multiple comparative analyses across family members increases the risk of false positive results, therefore, findings related to specific family members should be interpreted with caution. Future studies should incorporate extended follow-ups, objective measures, and multilevel designs to better understand the sustained processes of health behavior change.

## Conclusions

In summary, the comprehensive intervention enhanced family support, which subsequently mediated the improvement of children’s dietary habits and screen time. Future studies should incorporate family support into prevention and control strategies to improve intervention effectiveness.

## Supporting information

S1 Data CONSORT 2010 checklist of of the project. (DOCX)

S1 Table Description of the Multifaceted Intervention Components. (PDF)

S1 File Study protocol in English. (PDF)

S2 File Study protocol in Chinese. (PDF)
