# Supplementary material for: The effect of comprehensive intervention on family support and the mediating effect between intervention and changes in children’s dietary and physical activity behaviors
Source: PLoS One. 2026 Jan 22;21(1):e0339009. doi: 10.1371/journal.pone.0339009 (PMC12826510; doi:10.1371/journal.pone.0339009)
Supplement: S1 Data — (DOCX) [file pone.0339009.s001.docx]

**CONSORT 2010 checklist**

| ****Section/topic and item No**** | ****Standard checklist Item**** | **Corresponding Content in the Article** |
| --- | --- | --- |
| ****Title and Abstract**** | | |
| 1a | Identification as a randomised trial in the title | ****Title:**** The effect of comprehensive intervention on family support and the mediating effect between intervention and changes in children's dietary and physical activity behaviors |
| 1b | Structured abstract (design, methods, results, conclusions) | The abstract includes the trial design (cluster-randomized control), methods (questionnaire surveys, generalized estimating equation models, mediation analysis), results (higher family support in the intervention group, mediating effect of family support on dietary and screen time behaviors), and conclusions (comprehensive intervention is effective, family support plays a mediating role) |
| ****Introduction**** | | |
| **Background and objectives:** | | |
| 2a | Scientific background and explanation of rationale | See introduction section in text |
| 2b | Specific objectives or hypotheses | To investigate the effect of a comprehensive intervention on family support and to elucidate its mediating role in improving children's diet and physical activity, while also delineating the specific roles of different family members. |
| ****Methods**** | | |
| **Trial design:** | | |
| 3a | Description of trial design (such as parallel, factorial) including allocation ratio | Description of cluster-RCT design, allocation ratio (1:1) |
| 3b | Important changes to methods after trial commencement (such as eligibility criteria), with reasons | No changes |
| **Participants:** | | |
| 4a | Eligibility criteria for participants | Fourth-grade students who completed baseline and follow-up assessments. For detailed criteria , refer to the trial protocol. |
| 4b | Settings and locations where the data were collected | Changzhi, Shanxi Province, China, |
| **Interventions:** | | |
| 5 | The interventions for each group with sufficient details to allow replication, including how and when they were actually administered | The intervention group received five key components based on the socio-ecological model; the control group received no specific obesity intervention. See intervention section in text and S1 File in Supplementary information |
| **Outcomes:** | | |
| 6a | Completely defined prespecified primary and secondary outcome measures, including how and when they were assessed | primary outcome:the change in family support. |
| 6b | Any changes to trial outcomes after the trial commenced, with  reason | No changes |
| **Sample Size:** | | |
| 7a | How sample size was determined | BMI:MD=0.5, SD=1.4,α=0.05, 1-*β*=0.88, attrition rate=10% |
| 7b | When applicable, explanation of any interim analyses and stopping  guidelines | Not applicable |
| ****Randomization**** | | |
| **Sequence generation:** | | |
| 8a | Method used to generate the random allocation sequence | a centralized computer-generated randomization process |
| 8b | Type of randomisation; details of any restriction (such as blocking Details of stratification or matching if used and block size | Cluster randomization |
| **Allocation concealment mechanism:** | | |
| **9** | Mechanism used to implement the random allocation sequence  (such as sequentially numbered containers), describing any steps  taken to conceal the sequence until interventions were assign | Following baseline surveys, we distributed the schools equally between intervention and control arms (four per condition). Randomization was generated by means of a centralized computer, and an independent statistician maintained the concealed assignment sequences. |
| **Implementation:** | | |
| **10** | Who generated the random allocation sequence, who enrolled participants, and who assigned participants to interventions | **Generation of the random allocation sequence**:A centralized computer-generated randomization process;  **Enrollment of participants:** Participants were enrolled by local researchers after they had been assessed for eligibility and had provided informed consent;  **Assignment of participants to interventions:** An independent statistician maintained concealed allocation sequences to ensure blinding integrity. |
| **Blinding:** | | |
| 11a | If done, who was blinded after assignment to interventions (for  example, participants, care providers, those assessing outcomes) and how | outcome assessors |
| 11b | If relevant, description of the similarity of interventions | - |
| **Statistical Methods:** | | |
| 12a | Statistical methods used to compare groups for primary and secondary outcomes | Generalized estimating equation models |
| 12b | Methods for additional analyses, such as subgroup analyses and  adjusted analyses | subgroup analyses |
| ****Results**** | | |
| **Participant flow (a diagram is strongly recommended):** | | |
| 13a | For each group, the numbers of participants who were randomly assigned, received intended treatment, and were analysed for the primary outcome | The participant enrollment process is summarized in Figure 1. Initially, 400 fourth-grade students from eight recruited schools were enrolled in the baseline assessment. During the study period, four students (two from each group) were lost to follow-up due to illness, resulting in a final sample of 396 subjects (198 per group), with equal gender distribution (198 boys and 198 girls) |
| 13b | For each group, losses and exclusions after randomisation, together with reasons | During the study period, four students (two from each group) were lost to follow-up due to illness. See Fig 1 |
| Recruitment: | | |
| 14a | Dates defining the periods of recruitment and follow-up | September 2018- June 2019 |
| 14b | Why the trial ended or was stopped | Not applicable |
| **Baseline data:** | | |
| 15 | A table showing baseline demographic and clinical characteristics for each group | Table 1 Baseline demographic and anthropometric characteristics of study participants |
| Numbers analysed: | | |
| 16 | For each group, number of participants (denominator) included in each analysis and whether the analysis was by original assigned groups | Yes, see Table 2 |
| **Outcomes and estimation:** | | |
| 17a | For each primary and secondary outcome, results for each group, and the estimated effect size and its precision (such as 95% confidence interval) | Table 2 |
| 17b | For binary outcomes, presentation of both absolute and relative effect sizes is recommended | Table 2 |
| Ancillary analyses | | |
| 18 | Results of any other analyses performed, including subgroup analyses and adjusted analyses, distinguishing prespecified from exploratory | Subgroup analyses:  see Table 2-5 |
| Harms: | | |
| 19 | Important harms or unintended effects in each group (for specific  guidance see CONSORT for harms106) | No |
| ****Discussion**** | | |
| **Limitations:** | | |
| 20 | Trial limitations, addressing sources of potential bias, imprecision,and, if relevant, multiplicity of analyses | See the limitation section in this article |
| Generalisability: | | |
| 21 | Generalisability (external validity, applicability) of the trial findings |  |
| Interpretation: | | |
| 22 | Interpretation consistent with results, balancing benefits and harms,and considering other relevant evidence | the comprehensive intervention enhanced family support , which subsequently mediated the improvement of children's dietary habits and screen time. |
| **Other information** | | |
| **Registration:** | | |
| 23 | Registration number and name of trial registry | ClinicalTrials.gov NCT03665857, 11/09/2018 |
| Protocol: | | |
| 24 | Where the full trial protocol can be accessed, if available | refer to the Trial Protocol |
| **Funding:** | | |
| 25 | Sources of funding and other support (such as supply of drugs), role of funders | This research was supported by National Key R&D Program of China (2016YFC1300204). |
