# Supplementary material for: The effect of comprehensive intervention on family support and the mediating effect between intervention and changes in children’s dietary and physical activity behaviors
Source: PLoS One. 2026 Jan 22;21(1):e0339009. doi: 10.1371/journal.pone.0339009 (PMC12826510; doi:10.1371/journal.pone.0339009)
Supplement: S1 Table — (PDF) [file pone.0339009.s002.pdf]

Table 1. Description of the Multifaceted Intervention Components

Intervention components		Who delivered	When delivered	What delivered
**Three components targeting children**				
1. Health education		Trained class teachers	Every 2 to 3 weeks (10 sessions§)	Five core messages: two "*NOT*", two "*LESS*", and one "*MORE*" messages* were lectured on class
2.physical exercise reinforcement		Trained physical education teachers	Every school day	One-hour moderate-to-vigorous-intensity physical activity per day within school
3.BMI monitoring and feedback		Trained health care teachers	Monthly	Monitoring body weight and height of the children, and providing feedback on BMI status and changes
		Children	Weekly	Measuring body weight only
**Two components targeted the children's environment**				
schools	1.School policies supporting obesity prevention	Trained school teachers&	Every school day	ΨNot selling, eating, or buying unhealthy snacks or sugar-sweetened beverages within school
	2. Health education for school teachers	Trained project staff	In the first month (1 session)	Five core messages
families	1. Health education for parents	Trained project staff	In the start and halfway of the 1st semester, in the start of the 2nd semester (3 sessions)	Five core messages; feedback on children's BMI and behaviors through app
	2. Reinforcement of children's physical activity outside school	Parents	Every day	Supervising and encouraging children to perform physical activities outside of school
	3. Supporting children to manage body weight	Parents	Weekly	Recording and tracking diet and physical activity behaviors of the children (weekly) through app
			Monthly	Tracking BMI of the children (monthly) through app

*NOT eating excessively; NOT drinking sugar-sweetened beverages; LESS high-energy food; LESS sedentary time; MORE physical activities. & School teachers included school principals, class teachers, health care teachers, and physical education teachers. Ψ "Not selling": Not selling unhealthy snacks or sugar-sweetened beverages within school; "Not eating": Telling students not to eat unhealthy snacks or drink sugar-sweetened beverages within school; "Not buying": Students being educated by class teachers not to buy unhealthy snacks or sugar-sweetened beverages around school.
