# Supplementary material for: The effect of comprehensive intervention on family support and the mediating effect between intervention and changes in children’s dietary and physical activity behaviors
Source: PLoS One. 2026 Jan 22;21(1):e0339009. doi: 10.1371/journal.pone.0339009 (PMC12826510; doi:10.1371/journal.pone.0339009)
Supplement: S1 File — (PDF) [file pone.0339009.s003.pdf]

Technical Proposal

(I) Project Background

With economic development and lifestyle changes, the prevalence of overweight and obesity among children and adolescents in China continues to rise. This not only affects their current health but may also have long-term impacts on their future well-being and quality of life. Overweight and obesity during adolescence are closely associated with adult obesity and various chronic diseases. However, globally, there remains a lack of scalable intervention technologies targeting overweight and obesity in adolescent populations.

(II) Project Objectives

To develop a school-based intervention package for childhood overweight and obesity and rigorously evaluate its effectiveness and cost-effectiveness. Specific objectives include:

1. **Primary Objective**: To determine whether the average increase in Body Mass Index (BMI) among the intervention group is significantly lower than that of the control group.
2. **Secondary Objectives**:

(1) Assess the cost-effectiveness of the intervention technology.
(2) Determine whether the prevalence and incidence of overweight/obesity in the intervention group are significantly reduced compared to the control group.
(3) Evaluate whether the average increases in waist circumference, waist-to-hip ratio, blood pressure, and body fat percentage are significantly lower in the intervention group.
(4) Examine whether the proportion of children adopting healthy dietary and physical activity behaviors increases significantly in the intervention group.
(5) Assess whether the average physical fitness test scores improve significantly in the intervention group.
(6) Safety evaluation: Ensure no significant increase in the prevalence of underweight children in the intervention group.

(III) Project Design

1. Target Population

(1) Participating Schools: 24 primary schools (8 each from Beijing, Changzhi City in Shanxi, and Urumqi City in Xinjiang) meeting the following criteria: ①School leadership willing to cooperate, with supporting staff (e.g., school nurses, physical education teachers); ②At least 60 Grade 4 students (selected for literacy and follow-up feasibility), with 30-50 students per class; ③Even distribution across administrative districts.

Exclusion Criteria: ①Boarding, specialty, or minority schools; ②participation in other obesity-related programs within the past or upcoming year; ③plans for closure or relocation within two years.

(2) **Participating Classes**: 1-2 classes per school (minimum 40 students per class).

(3) **Participating Students**: All students with signed informed consent, excluding those with: ①Major organ diseases (e.g., heart disease, diabetes); ②Obesity due to other causes (e.g., endocrine disorders); ③Abnormal physical development, such as dwarfism, gigantism, etc; ④Physical developmental abnormalities or disabilities; ⑤Inability to participate in physical activities; ⑥Recent use of weight-loss medications or purging behaviors.

2. **Baseline Survey**

The baseline survey will be conducted in September 2018 prior to randomization. The baseline survey consists of three components: physical examinations, fitness tests, and questionnaires.

(1) **Physical Examinations**: Height, weight, waist/hip circumference, blood pressure, body composition (20-30 minutes per student). Accelerometers worn for one week in Beijing schools.

(2) **Fitness Tests**: 1-minute rope skipping, sit-ups, standing long jump, and 50m×8 shuttle run.

(3) **Questionnaires**: Student dietary/physical activity behaviors, parental involvement, and school obesity prevention policies.

3. **Randomization**

Schools stratified by administrative district and randomly assigned to intervention (12 schools) or control groups (12 schools).

(IV) Intervention Measures

Intervention Group: One academic year (September 2018–June 2019) of interventions, with follow-up until June 2020.

Control Group: No intervention during the project; materials provided post-study.

1. **Student Interventions**

(1)Health Education: 10 sessions (every 2-3 weeks) covering healthy weight knowledge and behaviors (e.g., "Two No's, Two Less's, One More": no overeating, no sugary drinks, less high-energy foods, less sedentary time, more play and activity).

(2)Enhanced Physical Activity:

In-school: 1 hour/day (moderate-to-vigorous intensity).

At-home: 30 minutes/weekday, 1 hour/weekend.

(3)Weight Monitoring: Monthly measurements by school nurses; weekly self-measurements recorded by students.

2. Parent Interventions

(1)Health Education Workshops: Semesterly lectures on healthy weight and mobile app usage (BMI tracking, behavior questionnaires).
(2)Home Activity Promotion: App reminders for parents to encourage children's exercise.

3. School Interventions

(1)Policy Implementation: Schedule integration, guaranteed activity time, restrictions on unhealthy snacks.
(2)Environment Building: Posters, improved food environment labeling.
(3)Teacher Training: Health education workshops and resource distribution.

4. Mobile App Functions

Knowledge dissemination, weight management, behavior monitoring, and automated feedback (BMI trends and recommendations).

(V) Follow-Up Surveys

Conducted at 4 months (January 2019), 9 months (June 2019), and 21 months (June 2020) post-baseline, mirroring baseline content (examinations, fitness tests, questionnaires).

Table 1. Baseline and Follow-Up Survey Content

**Component**	**Baseline (Sep 2018)**	**4-month (Jan 2019)**	**9-month (Jun 2019)**	**21-month (Jun 2020)**
**Physical Examinations**:				
Height	✓	✓	✓	✓
Weight	✓	✓	✓	✓
Waist circumference	✓	✓	✓	✓
Hip circumference	✓	✓	✓	✓
Blood pressure	✓	✓	✓	✓
Body composition	✓	✓	✓	✓
Accelerometer wear*	✓	✓	✓	✓
**Fitness Tests**				

**Component**	**Baseline (Sep 2018)**	**4-month (Jan 2019)**	**9-month (Jun 2019)**	**21-month (Jun 2020)**
1-minute rope skipping	✓		✓	
1-minute sit-ups	✓		✓	
Standing long jump	✓		✓	
Endurance run (50m×8)	✓		✓	
**Questionnaires**				
Health screening form*	✓			
Student questionnaire	✓		✓	
Parent questionnaire	✓		✓	
School questionnaire	✓		✓	

*Note: Accelerometers limited to Beijing schools; health screening questionnaire at baseline only.

Students will bring both the informed consent form and health screening questionnaire to their parents/guardians. Parents must provide consent for their child's participation in the project before completing the health screening questionnaire. Students who meet any of the exclusion criteria in the health screening will not participate in subsequent questionnaires, physical examinations, or fitness tests.
