# Supplementary material for: The effect of comprehensive intervention on family support and the mediating effect between intervention and changes in children’s dietary and physical activity behaviors
Source: PLoS One. 2026 Jan 22;21(1):e0339009. doi: 10.1371/journal.pone.0339009 (PMC12826510; doi:10.1371/journal.pone.0339009)
Supplement: S2 File — (PDF) [file pone.0339009.s004.pdf]

一、技术方案

（一）项目背景

随着经济发展以及生活方式的改变，我国儿童青少年的超重、肥胖率不断增加，不仅影响孩子现在的健康，对其日后的健康和生活质量也会造成影响。青少年时期的超重肥胖还与其成年后的超重肥胖以及多种重大慢性病密切相关。然而，全世界范围内目前仍缺乏针对青少年的、可大范围推广的超重肥胖群体干预技术。

（二）项目目的

开发一种基于学校的儿童超重肥胖群体干预技术包，并采取严格的评价方法评估其干预效果和成本效果。具体目标如下：

1.主要目标：干预组儿童平均体重指数增长量是否显著低于对照组。

2.次要目标

（1）干预技术的实施是否具有成本效果；

（2）与对照组相比，干预组儿童的超重肥胖率及新发超重肥胖率在干预后是否显著降低；

（3）与对照组相比，干预组儿童的平均腰围、腰臀比、血压和体脂百分比的增长是否有显著减少；

（4）与对照组相比，干预组儿童采取健康饮食与运动行为的比例在干预后是否有显著提高；

（5）与对照组相比，干预组儿童的体质测试平均成绩是否有显著提高；

（6）作为安全性评价，与对照组相比，干预组儿童的消瘦率在干预后是否显著增加。

（三）项目设计

1.项目对象

（1）入选学校：本项目将在北京、山西长治市和新疆乌鲁木齐市三个地区选择符合条件的小学开展。采用方便抽样的方式在三个地区各选择 8 所小学，共 24 所小学。由项目人员向当地教育部门和学校进行沟通，了解当地小学数量及各校学生人数、参加项目意愿等情况，根据学校纳入和排除标准确定入选的学校。

学校纳入标准：①学校领导愿意配合,有项目工作需要的人员（如校医/保健老师、体育老师）；②研究开始时四年级学生（选择四年级学生的原因是考虑该年级学生识字较好，可以更好地接受健康教育课传授的知识，同时，该年级段是两年随访时依然在小学的最高年级）人数不少于 60 人，每个班人数 30-50 人；③如每个地区的入选学校来自不同的行政区，每个行政区的学校数必须为偶数。

学校排除标准：①寄宿 、特长或少数民族学校；②学校近一年或下一年参加其他肥胖相关干预项目或活动；③学校在未来两年内有明确的撤销或搬迁计划。

（2）入选班级：项目学校选定之后，项目人员需进一步确定各学校参加项目的班级。班级能参加人数在 40 人以上的学校，可根据班主任的参加意愿选择其中意愿最好的一个班参加。如班级能参加人数不足 40 人，则需选择其中 2 个班级参加。如班级能参加人数不足 40 人且班级数量不足 2 个，则不能选择该学校，应另行选择符合条件的学校参加研究。

（3）入选学生：参加项目的学校和班级确定后，在基线调查开始前一周内，项目人员通过班主任向选定班级内的所有学生发放知情同意书，其中家长知情同意书让学生带回家给家长填写。所有签署了学生和家长知情同意书的学生均被邀请参加本项目。但其中符合以下排除标准的学生将在基线调查后退出本项目，他们将不参加之后的任何检查，他们的基线数据也不会用于干预效果的评估。如果他们在干预组学校，也不要求他们参与本项目的干预活动。

学生排除标准：①心、肺、肝、肾等重要脏器病史，如心脏病、高血压、糖尿病、肺结核、哮喘、肝炎、肾炎等；②由其它原因引起的肥胖，如内分泌疾病、药物副作用等；③身体发育异常，如侏儒症、巨人症等；④身体残缺、畸形，如严重脊柱侧弯、鸡胸、跛足、明显 O 型腿和 X 型腿等；⑤不能参加学校的体育活动（如胸痛、眩晕、骨/关节疼痛等）；⑥过去 1 个月内，曾有过催吐或服用减肥药等减肥行为。

2.基线调查

基线调查将在随机分组之前于2018年9月进行。基线调查的内容包括体检、体质测试和问卷调查三个部分。

（1）体检

由班主任和校医组织学生参加体检，由经过统一培训的项目组人员进行体检。

体检内容包括身高、体重、腰围、臀围、血压、体成分。体检约需 20-30 分钟/人。

在北京地区每个学校中任选一个班级，给学生佩戴便携式加速度计一周。

（2）体质测试

由班主任和体育老师组织参加项目的学生进行体质测试，包括 1 分钟跳绳、1 分钟仰卧起坐、立定跳远和耐力跑（50 米×8 往返跑）。

（3）问卷调查

学生问卷调查内容主要是饮食运动行为，家长问卷调查内容主要是与学生饮食运动有关的情况。学校问卷调查内容主要是学校肥胖防控的工作情况。其中，学生调查问卷由班主任协助项目组人员集中组织完成，家长问卷由学生带给家长填写后上交给班主任。

3.随机化分组

基线调查结束之后，所有学校将会被随机分为干预组和对照组（每组 12 个学校）。采用整群分层随机方法，按行政区进行分层，将 24 个学校随机分为干预校和对照校。

（四）干预措施

在整个项目期间只对干预组（12 所学校）的学校实施干预，干预时长为一个学年（2018 年 9 月—2019 年 6 月），但干预组和对照组均在基线调查后被随访 2 年。对照组学校在项目期间（2018 年 9 月-2020 年 6 月）不采取任何干预措施。在整个项目完成（2020 年 6 月最后一次随访）之后，对照组学校将得到项目相关的干预材料。

本项目在学生、家长和学校三个层面开展干预，包括健康教育、加强学生运动、制定与落实学校政策，同时应用手机 APP 促进各方沟通等措施。

1.针对学生的干预活动

（1）学生健康教育活动

活动的主要内容：学生健康教育活动的**核心信息**包括：健康体重的益处，如何测量和判定健康体重，如何达到健康体重（“两不两少一多”：不过量饮食、不喝含糖饮料、少吃高能量食物、少坐少宅、多玩多动）。

活动的主要形式：采用健康教育课和主题班会穿插的形式进行，每次 40 分钟。从 2018 年 9 月开始，每 2～3 周一次，共 10 次。其中，第一学期进行 6 次；第二学期进行 4 次。给项目班级的学生发放预防体重过快增长的科普书、学生营养状况评价盘。由接受过健康教育课培训的班主任或健康教育老师进行授课。

（2）加强学生运动

①加强学生在校运动

要求干预班级学生的在校运动时间（包括体育课、早操/课间操、课外活动）合计达到 1 小时/天，运动的强度达到中等以上。由体育老师组织，采用学生喜欢的、有趣味性的运动形式开展在校运动。要求每次课外活动至少做一个运动游戏。必要时进行分组活动。

②加强学生居家锻炼

要求学生增加居家锻炼时间，达到平日 30 分钟/天，周末 1 小时/天。利用手机 APP 给学生及家长提供平日版或周末版居家锻炼方案，提醒家长督促孩子多运动。鼓励学生多与家人、邻居或伙伴一起做运动游戏。

（3）体重监测

校医/保健老师每个月对干预校学生进行一次身高、体重测量，项目人员必须协助。同时，每个项目班级放置电子秤 1 个，由班级内学生每周自我测量一次体重并进行记录。

2.针对家长的干预活动

（1）家长健康教育活动

活动的主要内容：通过家长健康教育活动的核心信息包括：小学生健康体重的益处，小学生如何测量和判定健康体重，如何帮助孩子达到健康体重。通过家长健康教育活动教会家长使用手机 APP，动员家长配合完成：①查看推送的健康教育知识，支持孩子完成老师在健康教育活动中布置的“小手拉大手”活动；②定期填写手机 APP 中的饮食与运动行为问卷；③每月查看一次手机 APP 自动生成的学生“评估反馈”结果（包括学生体重和 BMI 情况及与历史 BMI 的对比、饮食和运动情况及与上月的对比和总体反馈结果及建议）。

活动的主要形式：每学期开展一次家长健康教育讲座，通知主要照顾学生生活的家长参加，约 30-40 分钟，由项目组专家授课，并发放相应指导材料。在第

一学期中期再开展一次家长座谈会，约 30-40 分钟，针对学生的体重和行为变化情况及遇到的困难或问题，以座谈会的形式进行面对面沟通和指导。如有必要，在第二学期再开展一次类似形式的家长座谈会。针对干预过程中出现的问题，项目人员在必要的时候需要联系家长进行沟通交流。

（2）督促学生加强居家锻炼

利用手机 APP 督促家长提醒孩子多运动。鼓励家长多与孩子一起做运动游戏。

3.针对学校的干预活动

（1）明确负责本项目的学校负责人，负责协调相关人员实施项目工作，明确责任分工。

（2）制定与落实以下相关政策：

①将项目班级的学生健康教育活动排入课表；

②保证在校运动时间（包括体育课、早操/课间操和课外活动）达到 1 小时.天，将需要额外增加的课外活动排入课表；

③安排项目班级家长健康教育讲座/座谈会和教师健康教育讲座；

④在全校范围（包括学校食堂、小卖部、自动贩卖机等地点），不建议售卖不健康零食和含糖饮料；学校内不允许食用不健康零食和含糖饮料；教育学生们不买校门口的不健康零食和含糖饮料。(“健康零食”是指在一日三餐以外吃的“奶制品、新鲜的蔬菜或水果、天然未加工的坚果类”这三类食物。**“不健康零食”**是指除了这三类以外的其他包装零食。)

（3）营造氛围

在参与项目的班级教室里定期张贴健康教育海报、改善学校食物环境标识。

（4）教师健康教育活动

干预初期组织学校项目工作组的相关教师及参与班级的任课教师参加的健康教育讲座，约 30-40 分钟，由项目组专家授课。给参加的教师发放正式出版的学生肥胖科普书、学生营养状况评价盘。

4. 应用手机 APP 促进各方联动

通过应用手机 APP 实现知识传播、体重管理、行为监测和评估反馈等功能，有效地促进项目人员-学校-家长-学生之间的联动。

（1）知识传播：

通过手机 APP 定期向家长和老师传播本项目健康教育活动的相关知识。

（2）体重管理：

根据每月体重监测信息（包括身高和体重），手机 APP 自动生成学生的体重目标（本次体重监测时的 BMI 在同年龄、同性别组 P50 及 P50 以上的学生有具体的体重目标），每月家长、老师和项目人员通过 APP 查看学生的体重变化和是否达到体重目标。

（3）行为监测：

每周家长通过手机 APP 填写学生饮食及运动行为问卷（反映学生在最近一周的饮食及运动行为），填写问卷后即可收到 APP 的自动反馈。

（4）评估反馈：

每月根据学生体重监测及行为监测信息，手机 APP 自动生成“评估反馈”结果，内容包括本次监测的 BMI 情况及与历史 BMI 的对比、饮食和运动情况及与上月的对比和总体反馈结果及建议，家长通过手机 APP 查看。项目人员和老师对班级情况进行评估及反馈。

（五）随访调查

计划在基线调查完成后 4 个月（2019 年 1 月）、9 个月（2019 年 6 月）和 21 个月（2020 年 6 月）对干预组和对照组学生同时进行随访调查。基线调查和三次随访调查的内容见**表** 1。

表 1 基线调查和三次随访调查的内容

内容	基线调查（2018.9）	4 个月随访（2019.1）	9 个月随访（2019.6）	21 个月随访（2020.6）
**体检：**				
身高	√	√	√	√
体重	√	√	√	√
腰围	√	√	√	√
臀围	√	√	√	√
血压	√	√	√	√
体成分	√		√	
佩戴加速度计*	√		√	
**体质测试**				
1 分钟跳绳	√		√	
1 分钟仰卧起坐	√		√	
立定跳远	√		√	
耐力跑	√		√	
**问卷调查：**				
健康筛查问卷#	√			
学生问卷	√		√	
家长问卷	√		√	
学校问卷	√		√	

*仅在北京项目学校完成佩戴加速度计；

#学生将知情同意书和健康筛查问卷一同带给家长，请家长知情同意参加本项目后再填写健康筛查问卷；符合健康筛查排除标准的学生将不参加之后的其他问卷调查、体检和体质测试。
